# Galbibacter kalidii sp. nov., a flexirubin-type pigment-producing bacterium, isolated from saline soil in Xinjiang

**DOI:** 10.1099/ijsem.0.007132

**Published:** 2026-05-12

**Authors:** Yin Huang, Yibo Yuan, Jinbiao Ma, Rashidin Abdugheni, Man Cai, Yongxia Wang, Wen-Jun Li, Li Li

**Affiliations:** 1 State Key Laboratory of Ecological Safety and Sustainable Development in Arid Lands, Xinjiang Institute of Ecology and Geography, Chinese Academy of Sciences, Urumqi 830011, PR China; 2 College of Life Science, Northwest A&F University, Yangling, Shaanxi 712100, PR China; 3 Department of Microbiology, School of Basic Medical Sciences, Xinjiang Medical University, Urumqi 830017, PR China; 4 China General Microbiological Culture Collection Center, Institute of Microbiology, Chinese Academy of Sciences, Beijing 100101, PR China; 5 Yunnan Institute of Microbiology, School of Life Sciences, Yunnan University, Kunming 650091, PR China; 6 State Key Laboratory of Biocontrol, Guangdong Provincial Key Laboratory of Plant Resources, School of Life Sciences, Sun Yat-sen University, Guangzhou 510275, PR China

**Keywords:** 16S rRNA gene, flexirubin-type pigment, *Galbibacter*, polyphasic taxonomy

## Abstract

A Gram-stain-negative, strictly aerobic, rod-shaped bacterium, designated EGI 63066^T^, was isolated from a saline soil of *Kalidium foliatum* (Pall.), collected from Wujiaqu in Xinjiang, China. Strain EGI 63066^T^ is a non-motile, rod-shaped bacterium; it forms pale yellow colonies on the marine agar and grows at 10–37 °C,at pH 6.0–9.0 and 0–10% (w/v) NaCl. The 16S rRNA sequences of the strain EGI 63066^T^ and *Galbibacter mesophilus* Mok-17^T^, *Robertkochia solimangrovi* CL23^T^, *Robertkochia sediminum* 1368^T^ and *Galbibacter pacificus* CMA-7^T^ share sequence similarities of 92.80%, 91.85%, 91.79% and 91.79%, respectively. Higher average amino acid identity values with *Galbibacter* (72.10–76.90%) than with *Robertkochia* (69.40–70.30%) supported the assignment of strain EGI 63066^T^ to the genus *Galbibacter*. Phylogenomic analysis based on core genes revealed that strain *Galbibacter kalidii* formed a stable clade with the four recognized species of the genus *Galbibacter* and the not validly published ‘*Joostella atrarenae*’ M1-2. The average nucleotide identity and digital DNA–DNA hybridization values between the strain EGI 63066^T^ and members of the genus *Galbibacter* were 71.10–74.15% and 18.00–19.70%, respectively. The DNA G+C content of the genome for strain EGI 63066^T^ based on genomic DNA was 39.0 mol%, and the genome size was 4.66 Mbp. The predominant fatty acids of strain EGI 63066^T^ were iso-C_15 : 0_, iso-C_15 : 1_ G and summed feature 3 (C_16 : 1_ *ω6*c and/or C_16 : 1_ *ω7*c). The major respiratory quinone was menaquinone-6, and the major polar lipids were phosphatidylethanolamine, aminolipid, aminophospholipid and phospholipid. Compared to other species within the genus *Galbibacter*, EGI 63066^T^ exhibits distinctive features, including the production of flexirubin-type pigments, CM-cellulose hydrolysis and whole-cell protein profiles. Based on the polyphasic taxonomic analysis, strain EGI 63066^T^ represents a novel species of the genus *Galbibacter*, for which the name *G. kalidii* sp. nov. is proposed. The type strain is EGI 63066^T^ (=JCM 36938^T^=CGMCC 1.19132^T^).

## Introduction

*Kalidium foliatum* (Pall.), commonly known as saltbush, is a small shrub species found in arid regions, including Heilongjiang, Inner Mongolia, northern Hebei, northern Gansu, Ningxia, Qinghai and Xinjiang in China [1]. This plant typically grows in saline-alkaline areas and along salt lake edges. Saltbush is a fleshy, succulent, salt-tolerant forage plant favoured by horses and sheep. It serves as a primary fodder source for camels. Its nutritional value is substantial for livestock in arid and desert regions during winter grazing and supplementary feeding [2]. Mining microbes from saltbush is valuable in understanding the profile and function of microbes associated with this salt-resistant plant.

The genus *Galbibacter* was proposed by Khan *et al*. [3]. At the time of writing, the genus *Galbibacter* only comprises four species with validly published names (https://lpsn.dsmz.de/genus/galbibacter, by 25 July 2025). Among them, the species *Joostella marina* was transferred into the genus *Galbibacter* and renamed as *Galbibacter orientalis* [4]. Species within the genus thrive in a wide range of salt concentrations ranging from 0 to 15% (w/v) [4] and are distributed in seawater [4] and marine sediment [3,5], respectively. The members are Gram-negative, catalase- and oxidase-positive bacteria, with menaquinone-6 (MK-6) as the major respiratory menaquinone, and the major fatty acids are iso-C_15 : 0_, iso-C_15 : 1_ G and C_16 : 1_ *ω6*c and/or C_16 : 1_ *ω7*c [3,5]. The genome size of *Galbibacter* ranges from 3.57 to 4.51 Mbp, and the genome G+C content ranges from 33.6 to 38.4 mol% [4].

Currently, the taxonomy of *Galbibacter* is limited due to the difficulty in obtaining culturable bacteria of rare groups by culture-dependent methods. Except for the marine environment, *Galbibacter* exists in compost, rumen and manure microbial communities that are detected using culture-independent methods [6,8]. It may play a crucial role in the mesophilic anaerobic processes for treating organic wastes and reducing contaminant loads. In the lower range of mesophilic temperatures (28 ℃), the *Galbibacter mesophilus* was found in the reactors [6]. However, knowledge of the function of *Galbibacter* is still scarce.

During the profiling and mining of salt-tolerant and plant-growth-promoting soil microbes of a halophytic plant, *K. foliatum*, collected from Wujiaqu in Xinjiang, China, we recovered many bacterial strains, which included some potential novel strains. In this study, we report the isolation, characterization and taxonomy of a novel bacterial isolate, strain EGI 63066^T^. Based on its phylogeny, genotypic and phenotypic properties, and distinct differences from related species within *Galbibacter*, strain EGI 63066^T^ is designated as a new species, and the name *Galbibacter kalidii* sp. nov. is proposed.

## Methods

### Isolation, cultivation and maintenance

The strain EGI 63066^T^ was isolated from bulk saline soil of *K. foliatum* (Pall.) collected at Wujiaqu (44° 13′ N 87° 40′ E), with sampling sites positioned 10–15 cm horizontally from the plant base at 10–20 cm depth (Table S2, available in the online Supplementary Material). To culture and isolate bacterial strains, 1.0 g of the fresh soil sample was diluted (10^−3^ to 10^−5^) using sterile distilled water, spread on M1 agar plates and incubated at 30 ℃ for 2 weeks. The M1 medium was composed of the following (per litre): yeast extract 0.25 g, K_2_HPO_4_ 0.5 g, NaCl 30.0 g and agar 20.0 g. Single colonies were picked and purified by continuous streaking. The purity was confirmed by the cleanness of the electropherogram obtained from 16S rDNA amplification and electrophoresis. The isolate was routinely cultured on marine agar (MA) 2216 [9] (Difco, China) at 30 ℃ and maintained as a 20% (w/v) glycerol suspension at −80 ℃.

### Phylogenetic and genome sequence analysis

Genomic DNA was extracted using the Takara MiniBEST Bacteria Genomic DNA Extraction Kit Ver. 3.0 (Takara Biotech Co. Ltd., Dalian, China), and the DNA extract was used as a template to amplify the 16S rRNA gene sequence by PCR using primers 8F (5′-AGAGTTTGATCCTGGCTCAG-3′) and 1492R (5′-GGTTACCTTGTTACGACTT-3′) [10], and the PCR products were used for 16S rRNA gene sequencing by SinoGenoMax Company (Beijing, China). The similarity of the 16S rRNA gene sequence was analysed using the EzBioCloud server (https://www.ezbiocloud.net/) [11]. We used 16S rRNA gene sequences of the strain EGI 63066^T^ and validly published type strains closely related with a similarity greater than 89.10% to construct phylogenetic trees. We first performed multiple sequence alignment using clustal W [12] and then constructed phylogenetic trees using the neighbour-joining (NJ) [13], maximum-likelihood (ML) [14] and maximum-parsimony (MP) [15] methods. Evolutionary distances were calculated using the algorithm of Kimura’s two-parameter model [16], and 500 bootstrap replications were performed using mega software (version 11.0) [17].

To further determine the phylogenomic relationships between EGI 63066^T^ and its closely related species, the genome of strain EGI 63066^T^ was sequenced using the HiSeq X platform (Illumina), and raw reads were assembled using SOAPdenovo (version 2.04) (http://soap.genomics.org.cn). A phylogenomic tree was reconstructed based on the genome sequences of strain EGI 63066^T^ and its phylogenetic neighbours, utilizing the 92 up-to-date bacterial core genes through the UBCG tree-building tool referring to previous reports [18,20]. The digital DNA–DNA hybridization (dDDH) and the average nucleotide identity (ANI) values between the draft genome sequences of strain EGI 63066^T^ and its phylogenetic neighbours were estimated using the Genome-to-Genome Distance Calculator (http://ggdc.dsmz.de/ggdc.php) [21], referring to the recommended formula. And the ANI Calculator uses the OrthoANIu algorithm (OrthoANIu) (https://www.ezbiocloud.net/tools/ani) [22], respectively. The percentage of average amino acid identity (AAI) was calculated using CompareM version 0.8.14 (https://github.com/dparks1134/CompareM), and percentages of conserved proteins (POCP) among strain EGI 63066^T^ and its phylogenetically closest neighbours were generated according to a previously reported method [23]. The genome of strain EGI 63066^T^ was annotated using the RAST Server (https://rast.nmpdr.org) [24].

### Morphology, biochemical and physiology

Cell morphology was determined using transmission electron microscopy (H7650, Hitachi) using cells grown on MA for 72 h. Gram staining, motility and catalase and oxidase activities were determined using the methods described previously by Dong and Cai [25]. Colony morphology was observed after incubation on MA at 30 ℃ for 48 h aerobically. There is a large amount of precipitation in the marine broth (MB) 2216 (Difco), so we used R2A liquid medium to determine growth tolerance to salinity at NaCl concentrations of 0, 1, 2, 3, 5, 7, 9, 10, 12, 15, 20 and 25% (w/v); tolerance of temperature at 4, 10, 20, 25, 30, 35, 37, 42 and 50 ℃ in the R2A liquid medium with 2% (w/v) NaCl; and pH ranges (4.0–13.0, 1.0 intervals). Cell cultures were incubated for 3 days at 37 ℃, by adjusting the pH before sterilization using buffers according to a previous report [26]. All the experiments mentioned above were carried out in triplicate. Hydrolysis of carboxymethyl cellulose (CM-cellulose), starch, casein, Tween 20 and tyrosine was tested in 2% (w/v) NaCl R2A medium supplemented with 1% (w/v) of different substrates, including CM-cellulose, soluble starch, skimmed milk, Tween 20 and tyrosine, respectively [27]. Degradation of DNA was tested by using Toluidine Blue DNAse Agar [28]. The flexirubin-type pigments were tested with 20% KOH (w/v) [29]. Anaerobic growth was tested by an anaerobic pouch (Thermo Scientific^™^ Oxoid AnaeroGen 2.5 L) on MA at 30 ℃ for 7 days. Other biochemical characteristics, enzyme activities, acid production and utilization of sole carbon and nitrogen sources were evaluated using API 20NE and API ZYM bacterial identification kits (bioMérieux, France) and the BIOLOG GEN III MicroStation system (BIOLOG, USA), respectively, following manufacturers’ instructions. The antibiotic susceptibility of EGI 63066^T^ to the following antibiotics was assessed by the disc diffusion method [30] by incubating the culture plates on MA for 48 h at 30 ℃. The antibiotics tested were the following antibiotics (micrograms per disc unless otherwise indicated): fleroxacin (5), lomefloxacin (10), ciprofloxacin (5), penicillin (10 IU), erythromycin (15), chloromycetin (30), azithromycin (15), clindamycin (15), doxycycline (30), clarithromycin (15), tobramycin (10), vancomycin (30), netilmicin (30), ceftriaxone (30), cefaclor (30), cefazolin (30), cefotaxime (30), ampicillin (10), cefuroxime sodium (30), minocycline (30), rifampicin (5), tetracycline (30), amikacin (30), ceftazidime (30), cephalothin (30), oxacillin (1), nitrofurantoin (300) and cefoperazone (75).

### Chemotaxonomy and whole-cell protein analysis

The whole-cell fatty acids of strains EGI 63066^T^ and *G. mesophilus* CGMCC 1.5663^T^ were extracted according to the standard protocol of the Sherlock Microbial Identification System (MIDI, TSBA6) [31], using harvested cells after incubation in MA at 30 ℃ for 72 h, and determined using gas chromatography (Agilent 6890 N; Hewlett Packard) and identified using the TSBA6.0 database of the Sherlock Microbial Identification System [32]. Cell biomass for analysis of respiratory quinones of strains EGI 63066^T^ and *G. mesophilus* CGMCC 1.5663^T^ was incubated in shaken flasks containing MB at 30 ℃, harvested at the same physiological age (initial stationary phase) and determined according to previously described methods [33,34]. Polar lipids were extracted and determined using two-dimensional TLC [33,35]. Whole-cell protein profiles of strains EGI 63066^T^ and *G. mesophilus* CGMCC 1.5663^T^ were analysed using MALDI-TOF MS microTyper MS (Tianrui, China) [31], and the spectra were exported to MT Master 1.0 software and IDBac (https://chasemc.github.io/IDBac/) for processing and evaluation by cluster analysis [36].

## Results and discussion

### Phylogenetic and genomic analyses

**Fig. 1.**
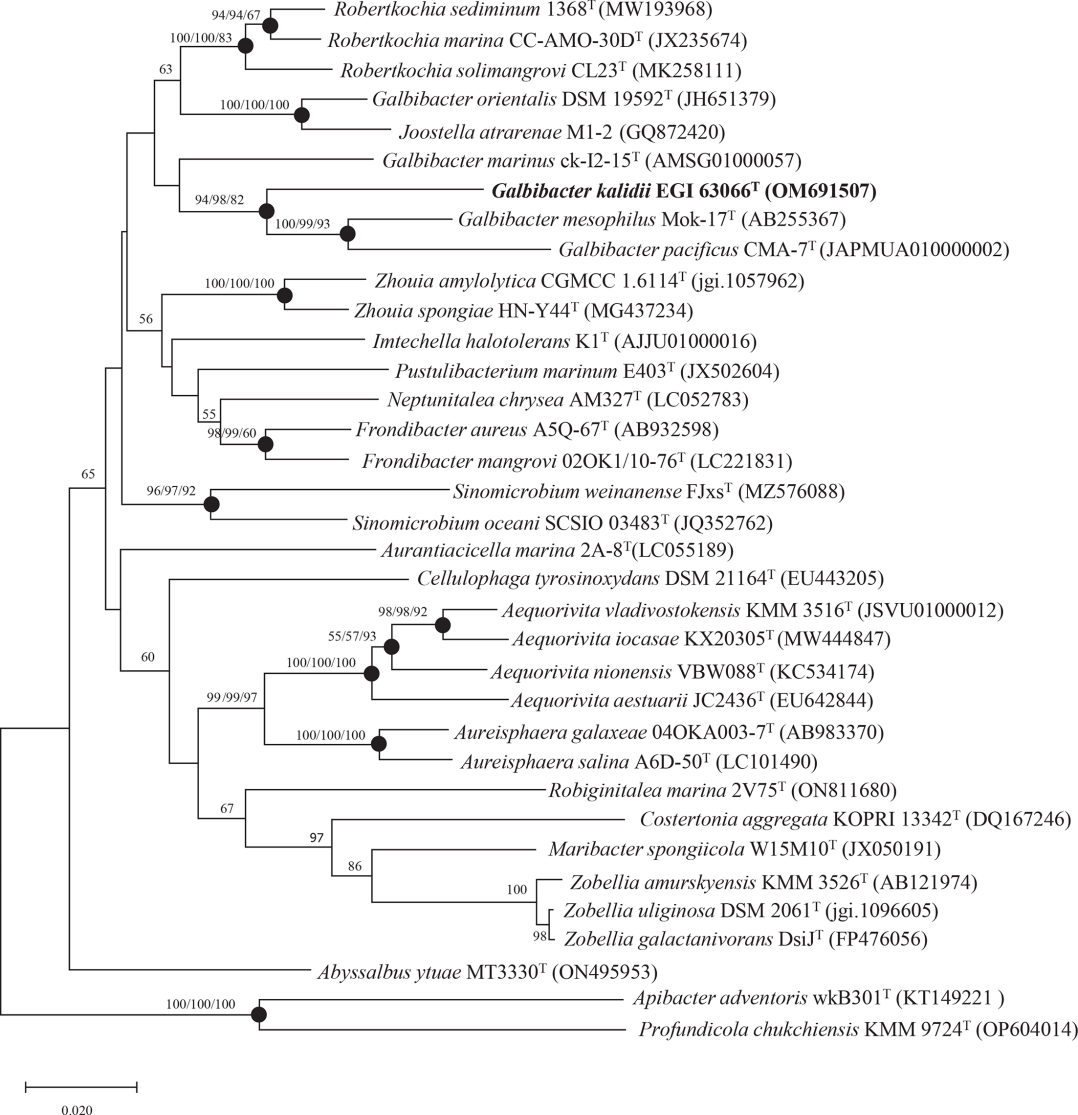
The NJ phylogenetic tree constructed based on 16S rRNA gene sequences of the strain EGI 63066^T^ and related validly published phylogenetic neighbours. This tree was reconstructed by using the NJ method with the algorithm of Kimura’s two-parameter model with 500 bootstraps. Filled circles indicate nodes overlapping on trees reconstructed by ML and MP algorithms. Numbers at nodes indicate the percentage of 500 bootstrap replicates. These bootstrap values are depicted in the order NJ/ML/MP. Only bootstrap values above 50% are shown. GenBank accession numbers are given in parentheses. Bar, 0.02 substitutions per nucleotide position. *Apibacter adventoris* wkB301^T^ and *Profundicola chukchiensis* KMM 9724^T^ were used as the outgroup. ‘*Joostella atrarenae*’ M1-2 (not validly published) is included for reference.

**Fig. 2.**
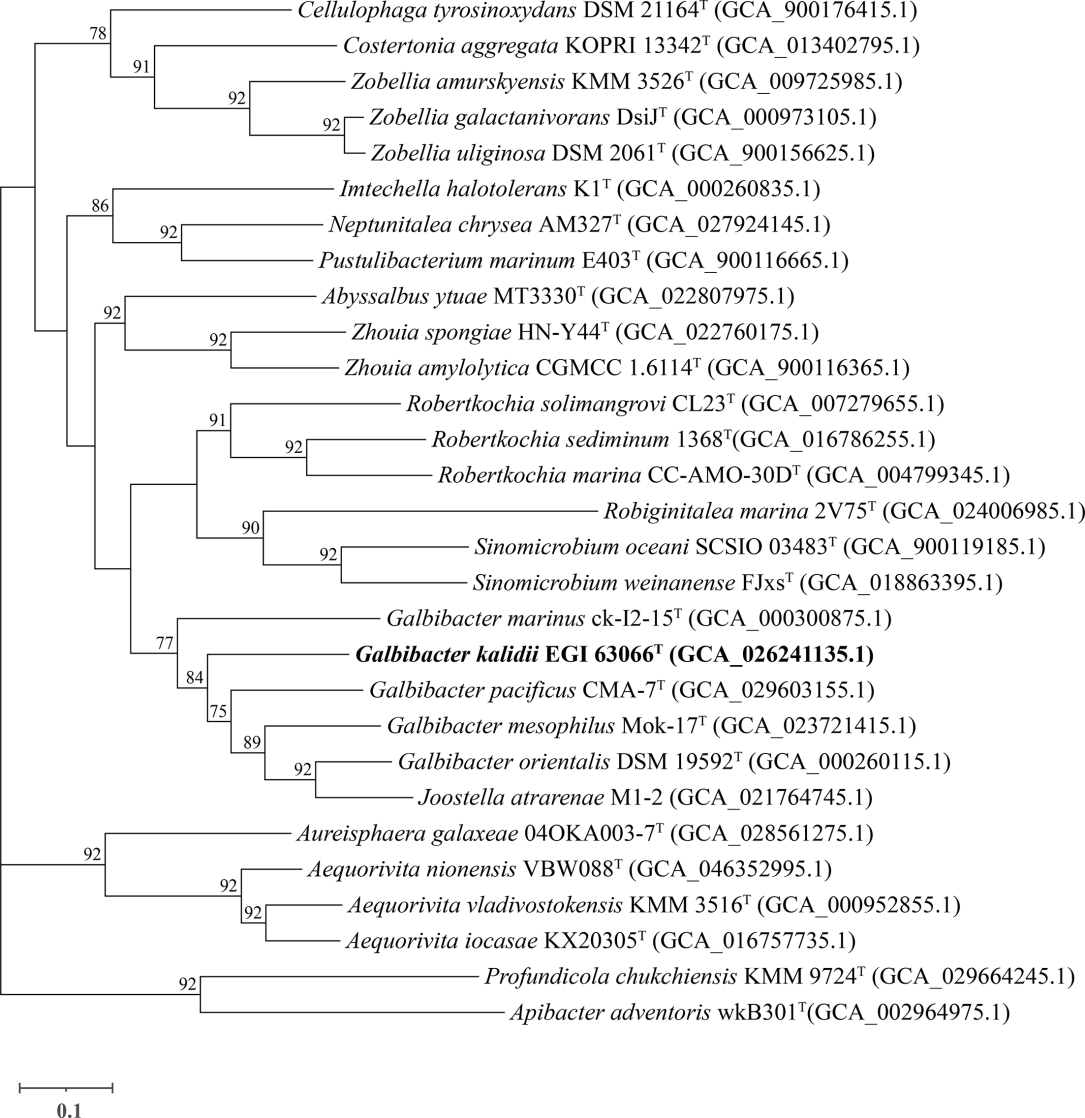
Phylogenomic tree constructed using 92 bacterial core gene sequences of the genomes of the strain EGI 63066^T^ and its closely related 26 strains available on NCBI GenBank. GenBank accession numbers are shown in parentheses. Gene support index (GSI) values of 92 UBCGs are given at branching points. Bar, 0.10 substitutions per position. ‘*Joostella atrarenae*’ M1-2 (not validly published) is included for reference.

The 16S rRNA gene sequence of strain EGI 63066^T^ (1,401 bp) is closely related to *G. mesophilus* Mok-17^T^ (92.80%), with the identities of 91.85%, 91.79%, 91.79%, 91.14%, 90.86% and 90.21% between *Robertkochia solimangrovi* CL23^T^, *Robertkochia sediminum* 1368^T^, *Galbibacter pacificus* CMA-7^T^, *Robertkochia marina* CC-AMO-30D^T^, *Galbibacter marinus* ck-I2-15^T^ and * G. orientalis* DSM 19592^T^, respectively. Lower sequence similarity values were shown to all other members of the family *Flavobacteriaceae* (<90.0%). The phylogenetic trees constructed using NJ, ML and MP techniques showed that strain EGI 63066^T^ formed a clade with *G. mesophilus* Mok-17^T^ and *G. pacificus* CMA-7^T^ (Fig. 1). The phylogenomic tree based on whole genomes was reconstructed (Fig. 2), showing that EGI 63066^T^ was closely clustered with the four established *Galbibacter* species and ‘*J. atrarenae*’ M1-2 with a high bootstrap value (77%) which proved that strain EGI 63066^T^ is a member of this genus. It should be noted that ‘*Joostella atrarenae*’ M1-2 is not a validly published name. Its phylogenetic position, however, suggests a close affinity with the genus *Galbibacter*, which may warrant future taxonomic revision upon valid publication. The AAI values between strain EGI 63066^T^ and other members of the genus *Galbibacter* (72.10–76.90%) were considerably higher than those between the strain and members of the genus *Robertkochia* (69.40–70.30%), providing robust genomic evidence supporting its assignment to the genus *Galbibacter* (Table S1). The dDDH and ANI values of strain EGI 63066^T^ with other members of the genus *Galbibacter* were 18.00–19.70% and 71.1–73.83% (Table 1), respectively. Considering that the dDDH and ANI values were well below the proposed threshold of 70% (dDDH) and 95% (ANI) for the delineation of bacterial species [37], denoting strain EGI 63066^T^ represents a novel species of the genus *Galbibacter*, from the genomic perspective. Genome sequencing results showed that pair-end sequencing (4,657,744 bp) of a fragment library created about 4,657,353 quality-filtered reads. Filtered reads were assembled into 113 scaffolds with a total sequence length of 4.7 Mbp (contig N50: 175,940 bp). The G+C content of the genome of strain EGI 63066^T^ was 39.0 mol%, which was similar to those of the closely related type strains *G. mesophilus* Mok-17^T^ (37.3%) and * R. solimangrovi* CL23^T^ (40.72%). The completeness and contamination of the genome assembly of strain EGI 63066^T^ were 99.01 and 0.77%, respectively, indicating that the genome assembly is of high genome quality. RAST annotation results showed that the strain contains 4,406 protein-coding genes and 43 RNA-coding genes (Fig. S1), with functional annotations revealing 125 genes associated with carbohydrate metabolism, 204 with amino acid metabolism, 152 with cofactor/vitamin/prosthetic group/pigment biosynthesis, 63 with nucleotide metabolism, 13 with nitrogen metabolism, 15 with phosphorus metabolism and 7 with potassium metabolism. Basic annotated metabolic pathways included pyruvate-alanine-serine interconversions, the pentose phosphate pathway, pyruvate metabolism I (anaplerotic reactions), pyruvate metabolism II (acetyl-CoA/acetogenesis), glycogen metabolism and tetrahydropterine-mediated one-carbon metabolism.

**Table 1.** The genome-based comparison of the strain EGI 63066^T^ and its phylogenetically related neighbours Strains: 1, *G. mesophilus* Mok-17^T^ (GCA_023721415.1) [3]; 2, *G. pacificus* CMA-7^T^ (GCA_029603155.1) [4]; 3, *G. orientalis* DSM 19592^T^ (GCA_000260115.1) [4]; 4, *G. marinus* ck-I2-15^T^ (GCA_000300875.1) [5]; 5, *A. ytuae* MT3330^T^ (GCA_022807975.1) [39]; 6, *R. solimangrovi* CL23^T^(GCA_007279655.1) [40]; 7, *R. sediminum* 1368^T^(GCA_016786255.1) [41]; 8, *R. marina* CC-AMO-30D^T^ (GCA_004799345.1) [42]. Notes: Calculations were conducted by comparing against the strain EGI 63066^T^, and the GenBank accessions were given in parentheses.

Strain	Genome length (Mbp)	G+C content (mol%)	Pairwise ANI value (%)	dDDH value (%)	AAI (%)	POCP (%)	G+C difference (in mol%)
**1**	3.77	37.27	73.52	19.70	76.70	60.70	1.73
**2**	4.04	38.38	74.15	19.20	76.80	64.30	0.61
**3**	4.51	33.59	73.83	19.50	76.90	62.10	5.40
**4**	3.57	37.04	71.1	18.00	72.10	61.40	1.95
**5**	4.37	35.22	70.81	20.40	69.40	54.40	3.77
**6**	4.41	40.72	70.35	18.70	70.30	56.40	1.72
**7**	3.53	45.74	70.01	18.40	69.40	52.60	6.74
**8**	3.58	43.63	69.96	18.50	69.80	53.00	4.63

### Morphological, physiological and chemotaxonomic characteristics

**Fig. 3.**
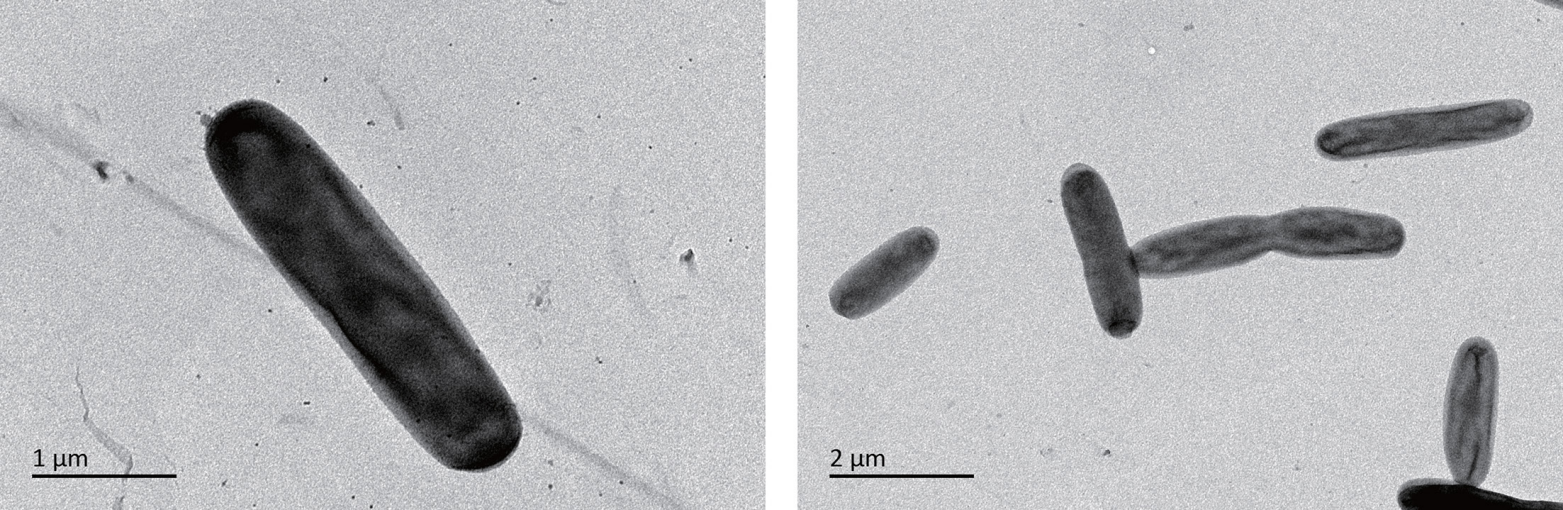
Cell morphology of the strain EGI 63066^T^. Bar, 1 µm (left panel); bar, 2 µm (right panel).

Strain EGI 63066^T^ was non-motile, Gram-stain-negative, strictly aerobic, rod-shaped (0.78–0.84×2.6–3.12 µm) (Fig. 3). Colonies were pale yellow, circular, opaque and smooth after incubation on MA at 30 ℃ for 48 h (Fig. S2). Growth was observed at 10–37 ℃ (optimum 25 ℃), at pH 6.0–9.0 (optimum pH 7.0) and NaCl concentrations of 0–10% (w/v; optimum 3%). Strain EGI 63066^T^ was resistant to fleroxacin, lomefloxacin, penicillin, chloromycetin, doxycycline, tobramycin, vancomycin, netilmicin, cefazolin, cefotaxime, ampicillin, cefuroxime sodium, minocycline, amikacin, cephalothin and oxacillin but sensitive to ciprofloxacin, erythromycin, azithromycin, clindamycin, clarithromycin, ceftriaxone, cefaclor, rifampicin, tetracycline, ceftazidime, cefoperazone or nitrofurantoin.

Furthermore, strain EGI 63066^T^ exhibits positive reactions for oxidase, catalase, tyrosine, casein, Tween 20, CM-cellulose, aesculin hydrolysis, alkaline phosphatase, esterase (C4), esterase lipase (C8), leucine arylamidase, valine arylamidase, cystine arylamidase, trypsin, acid phosphatase, *α*-galactosidase, *β*-galactosidase, *β*-glucuronidase, *α*-glucosidase, *β*-glucosidase, *N*-acetyl-*β*-glucosaminidase, *α*-mannosidase, *β*-fucosidase and *α*-chymotrypsin. In addition, strain EGI 63066^T^ utilizes diverse substances for its growth, such as d-fructose, l-rhamnose, d-raffinose, *α*-d-glucose, *α*-d-lactose, d-mannose, d-maltose, d-melibiose, d-arabitol, d-trehalose, d-galactose, d-cellobiose, d-salicin, d-fucose, l-fucose and d-serine, indicating its ability to hydrolyse and convert various substrates.

Strains EGI 63066^T^ and *G. mesophilus* CGMCC 1.5663^T^ were positive for oxidase, catalase, hydrolysis of casein, Tween 20 and starch (weakly) and negative for degradation of DNA. However, EGI 63066^T^ was positive for CM-cellulose hydrolysis while * G. mesophilus* CGMCC 1.5663^T^ was negative. The comparative features of strain EGI 63066^T^ and the other type strains of the genus *Galbibacter* were presented in Table 2, denoting the differences between strain EGI 63066^T^ and four related *Galbibacter* species.

**Table 2.** Characteristics that differentiate strain *G. kalidii* EGI 63066^T^ from the closely related species Strains: 1, EGI 63066^T^; 2, *G. mesophilus* CGMCC 1.5663^T^; 3, *G. pacificus* CMA-7^T^; 4, *G. marinus* ck-I2-15^T^; 5, *G. orientalis* DSM 19592^T^. Data of strains 1 and 2 are from the current study, and data of strains 3–5 were from previously published reports [4,5, 43].

Characteristic	1	2	3	4	5
Isolation source	Saline soil	Coastal sea sediment	Ocean surface seawater	Deep-sea sediment	Coastal seawater
Colony morphology	Pale yellow	Yellow	Yellowish	Brownish-yellow	Bright-yellow
Cell length (µm)	2.6–3.1	1.0–3.0	1.9–2.7	1.7–2.9	1.0–2.0
Cell width (µm)	0.8–0.9	0.5–0.7	0.7–0.8	0.4–0.5	0.2–0.3
Temperature range of growth (°C)	10–37 (25)	10–42 (25)	10–40 (28–32)	10–37 (25)	10–37 (30)
NaCl range for growth (optimum) (%, w/v)	0–10 (3)	0–10 (3)	0–9 (3)	0–9 (1–3)	0–15 (1–3)
pH range (optimum) for growth	6–9 (7)	6–10 (8)	6–8 (7)	6–10 (7–8)	5.3–10.5 (5.3–7.6)
Flexirubin-type pigments	+	−	−	−	−
**Hydrolysis of:**					
Casein	w	w	+	−	−
CM-cellulose	+	−	nd	nd	nd
DNA	−	−	nd	−	nd
Starch	+	+	+	−	+
Tween 20	+	+	−	nd	nd
Tyrosine	w	+	nd	nd	nd
Aesculin	+	+	+	−	+
Gelatin	−	＋	+	−	−
Reduction of nitrate	−	+	+	+	−
Reduction of nitrite	−	−	+	−	−
**Enzyme activities (API ZYM):**					
Lipase (C14)	−	−	−	w	−
*α*-Chymotrypsin	w	w	+	+	−
*α*-Galactosidase	+	+	+	+	−
*β*-Glucuronidase	+	+	−	+	+
Naphthol-AS-BI-phosphohydrolase	−	−	+	+	+
*β*-Fucosidase	+	+	−	−	−
**Assimilation of:**					
Urease	−	+	−	−	−
*β*-Galactosidase	+	+	−	+	+
d-Glucose	+	−	w	+	+
l-Arabinose	−	−	w	+	+
d-Mannose	+	−	w	−	+
*N*-Acetylglucosamine	−	−	−	+	−
d-Maltose	+	−	+	−	+
Malic acid	+	−	w	−	−
Trisodium citrate	−	+	−	−	−
Polar lipid profile*	PE, AL, APL, PL, L	PE, AL, L	PE, AL, APL, PL, PGL, GL, L	PE, PL, AL, GL, L	PE, AL, L
DNA G+C content (mol%)	39.0	37.3	38.4	37.0	33.6

Symbols: *, polar lipid profile abbreviations defined herein; +, positive; −, negative; w, weakly positive; PE, phosphatidylethanolamine; AL, aminolipid; APL, aminophospholipid; PL, phospholipid; PGL, phosphoglycolipid; GL, glycolipid; L, unidentified lipids; nd, no data.

Note: All five bacterial strains are Gram-negative rods capable of growth in saline to hypersaline environments. They consistently utilize cellobiose as carbon sources and exhibit starch hydrolysis activity. Enzymatically, these strains demonstrate positive reactions for *α*-glucosidase, *β*-glucosidase, *α*-mannosidase, alkaline phosphatase, esterase (C4), esterase lipase (C8), leucine arylamidase, valine arylamidase, cystine arylamidase, trypsin and *β*-galactosidase. Furthermore, MK-6 serves as the predominant respiratory quinone across all isolates.

The predominant fatty acids (≥10%) of strain EGI 63066^T^ were iso-C_15 : 0_, iso-C_15 : 1_ G and summed feature 3 (C_16 : 1_ *ω6*c and/or C_16 : 1_ *ω7*c), which were similar to the other four reference strains (Table 3), but their proportions varied. The major respiratory quinone was MK-6, which was the major quinone of all current members of the genus *Galbibacter*. The polar lipids of strain EGI 63066^T^ were phosphatidylethanolamine, two aminolipids, aminophospholipid, phospholipid and unidentified polar lipids (Fig. S3), which were similar to those of the other type strains of the genus *Galbibacter* (Table 2). MALDI-TOF MS spectra analysis revealed the different whole-cell protein profiles of strain EGI 63066^T^ compared against its phylogenetically closest neighbour, strain CGMCC 1.5663^T^, and detailed results were presented in Fig. S4. For instance, major peaks at m/z 3113, 3422, 6231, 6445 and 6644 were observed in strain EGI 63066^T^ only, while the major peaks at m/z 2904, 3534, 6543, 6592 and 7068 were observed only in strain CGMCC 1.5663^T^. In addition, peaks at m/z 2298, 4598, 4665, 5313 and 6847 were identical between the two strains.

**Table 3.** Cellular fatty acid contents (%) of strain EGI 63066^T^ and the closest relatives of the genus *Galbibacter* Strains: 1, EGI 63066^T^; 2, *G. mesophilus* CGMCC 1.5663^T^; 3, *G. pacificus* CMA-7^T^; 4, *G. marinus* ck-I2-15^T^; 5, *G. orientalis* DSM 19592^T^. Data of strains 1 and 2 are from the current study, and data of strains 3–5 were from previously published reports [4,5, 43]. Fatty acids amounting to <1% of the total fatty acids in all strains are not shown. TR, trace (<1%); ‘–’, not detected.

Straight-chain	1	2	3	4	5
C_14 : 0_	1.89	TR	–	–	–
C_15 : 0_	–	–	–	–	5
C_16 : 0_	1.24	TR	2.0	2.2	2.0
C_18 : 0_	–	TR	TR	3.1	–
Unsaturated					
C_15 : 1_ *ω*6*c*	2.04	3.01	TR	TR	–
C_17 : 1_ *ω*8*c*	1.12	1.04	–	–	–
C_17 : 1_ *ω*6*c*	–	2	TR	TR	1.0
C_18 : 1_ *ω*5*c*	–	–	TR	1.3	–
C_18 : 1_ *ω*9*c*	TR	TR	–	1.7	–
C_20 : 1_ *ω*7*c*	1.38	TR	–	–	–
Branched					
iso-C_14 : 0_	–	3.39	–	–	–
iso-C_15 : 0_	37.6	15.47	24.7	20.5	14.5
iso-C_16 : 0_	1.13	1.77	1.8	TR	2.5
iso-C_17 : 0_	1.09	TR	–	–	–
iso-C_15 : 1_ G	19.02	12.47	11.2	7.5	9.8
anteiso-C_15 : 0_	1.33	TR	3.0	1.8	2.3
iso-C_16 : 1_ h	TR	1.47	1.0	TR	1.5
iso-C_17 : 0_ *ω*9*c*	–	–	–	–	12.5
Hydroxy					
iso-C_15 : 0_ 3-OH	8.72	7.17	–	4.6	3.2
C_15 : 0_ 2-OH	1.83	1.2	–	1.0	TR
C_15 : 0_ 3-OH	–	2.07	5.6	1.6	–
iso-C_16 : 0_ 3-OH	TR	1.3	TR	2.1	1.3
C_16 : 0_ 3-OH	–	TR	1.2	TR	1.4
iso-C_17 : 0_ 3-OH	–	21.38	18.9	15.7	13.3
Summed feature 3^*^	11.89	13.78	10.9	9.2	8.4
Summed feature 4^*^	–	–	TR	–	2.2
Summed feature 8^*^	TR	TR	–	4.7	–
Summed feature 9^*^	6.15	7.4	10.7	14.1	–
Unknown					
ECL 13.565	–	–	–	–	12.3

*Summed features are fatty acids that cannot be resolved reliably from another fatty acid using the chromatographic conditions chosen. The MIDI system groups these fatty acids as one feature with a single percentage of the total.

Summed feature 3 comprised C_16 : 1_* ω6*c and/or C_16 : 1_* ω7*c; summed feature 4 comprised iso-C_17 : 1_ I/anteiso-B and/or anteiso-C_17 : 1_ B/iso-I; summed feature 8 comprised C_18 : 1_ *ω*6*c* and/or C_18 : 1_ *ω*7*c*; summed feature 9 comprised C_16 : 0_ 10-methyl and/or iso-C_17 : 1_* ω9*c.

## Discussion and taxonomic conclusion

Identical to the specific features of *Galbibacter* members, we found that strain EGI 63066^T^ tolerates salinity at NaCl concentrations of 0–10% and grows at a wide range of temperatures (10–37 ℃), indicating that strain EGI 63066^T^ could survive under certain harsh conditions regarding the salinity and temperature. Being a strictly aerobic, salt-tolerant bacterium, strain EGI 63066^T^ could be developed as a model organism in a bioreactor composting system [7,38]. Additionally, we have found that this strain is sensitive to ciprofloxacin, erythromycin, azithromycin, clindamycin, clarithromycin, ceftriaxone, cefaclor, rifampicin, tetracycline, ceftazidime, cefoperazone and nitrofurantoin, which means strain EGI 63066^T^ could be a relatively safe candidate to be developed as an industrial cell factory.

Furthermore, we determined that the AAI values between *R. solimangrovi* CL23^T^ and *Galbibacter* strains EGI 63066^T^, *G. pacificus* CMA-7^T^ and *G. marinus* ck-I2-15^T^ were 70.3%, 70.5% and 70.2%, respectively. The *Robertkochia–Galbibacter* clade exhibits unusually high inter-genus AAI values, necessitating a customized genus delineation threshold of 70.50–72.10%.

Concurrently, AAI values between *R. solimangrovi* CL23^T^ and the type strains of *R. sediminum* 1368^T^ and *R. marina* CC-AMO-30D^T^ were only 70.00 and 70.20%. These values fall below the proposed genus delineation threshold (70.50–72.10%). Therefore, the low AAI values provide genomic evidence suggesting the potential for reclassifying *R. solimangrovi* into a novel genus. However, further evidence is required to substantiate this taxonomic revision.

In summary, strain EGI 63066^T^ is divergent from its phylogenetic relatives from genetic, genomic, chemotaxonomic and physiological perspectives. Genome-based analyses provided robust evidence for its taxonomic position. AAI comparisons revealed higher values with *Galbibacter* species (72.10–76.90%) than with *Robertkochia* species (69.40–70.30%), strongly supporting its assignment to the genus *Galbibacter*. Furthermore, phylogenomic analysis indicated that strain EGI 63066^T^ formed an independently stable clade with the four established *Galbibacter* species and ‘*Joostella atrarenae*’ M1-2. Moreover, ANIb and dDDH values between EGI 63066^T^ and reference type strains were lower than thresholds for distinguishing different species. In addition, in terms of the different whole-cell protein profiles and production of flexirubin-type pigments, strain EGI 63066^T^ is distinguished from its reference strains.

Conclusively, according to phylogenetic analysis and the comparison of the phenotypic and genomic characteristics, strain 63066^T^ can be assigned to a novel species of the genus *Galbibacter*, for which the name *G. kalidii* sp. nov. is proposed.

## Description of *Galbibacter kalidii* sp. nov.

*Galbibacter kalidii* (ka.li’di.i. N.L. gen. neut. n. *kalidii*, of the plant *K. foliatum*, indicating that the type strain was isolated from the soil of *K. foliatum*).

Cells are Gram-stain-negative, strictly aerobic, rod-shaped. Colonies on MA are pale yellow, circular and smooth after incubation for 48 h at 30 ℃. Cells grow at 10–37 ℃ (optimum 25 ℃), at pH 6.0–9.0 (optimum pH 7.0) and NaCl concentrations of 0–10% (w/v; optimum 3%). Cells are positive for oxidase, catalase, flexirubin-type pigments, degradation of starch, Tween 20 and CM-cellulose and weakly positive for degradation of tyrosine and casein. Cells are positive for alkaline phosphatase, esterase (C4), esterase lipase (C8), leucine arylamidase, valine arylamidase, cystine arylamidase, trypsin, acid phosphatase, *α*-galactosidase, *β*-galactosidase, *β*-glucuronidase, *α*-glucosidase, *β*-glucosidase, *N*-acetyl-*β*-glucosaminidase, *α*-mannosidase and *β*-fucosidase, and weakly positive for *α*-chymotrypsin, but negative for lipase (C14) and Naphthol-AS-BI-phosphohydrolase. Cells are able to utilize d-fructose, l-galactonic acid lactone, d-glucuronic acid, l-glutamic acid, sucrose, l-rhamnose, acetic acid, d-raffinose, *α*-d-glucose, *α*-d-lactose, d-mannose, glycyl-l-proline, d-maltose, d-melibiose, d-arabitol, d-lactic acid methyl ester, d-trehalose, *β*-methyl-d-glucoside, d-galactose, myo-inositol, d-gluconic acid, l-lactic acid, d-cellobiose, d-salicin, 3-methyl glucose, glycerol, l-aspartic acid, gentiobiose, d-fucose, *α*-keto-glutaric acid, acetoacetic acid, d-glucose-6-PO_4_, glucuronamide, *N*-acetyl-*β*-d-mannosamine, l-fucose, d-malic acid, d-turanose, *N*-acetyl-d-galactosamine, l-pyroglutamic acid, quinic acid, l-malic acid, stachyose, *N*-acetyl-neuraminic acid and 1% sodium lactate. Cells are resistant to aztreonam, rifamycin SV, lithium chloride, potassium tellurite, lincomycin, vancomycin, nalidixic acid, tetrazolium violet, d-serine and tetrazolium blue. The predominant fatty acids are iso-C_15 : 0_, iso-C_15 : 1_ G and summed feature 3 (C_16 : 1_ *ω6*c and/or C_16 : 1_ *ω7*c). The major respiratory quinone is MK-6. The major polar lipids are phosphatidylethanolamine, two aminolipids, aminophospholipid, phospholipid and unidentified lipids.

The type strain EGI 63066^T^ (=JCM 36938^T^=CGMCC 1.19132^T^) was isolated from the bulk soil of *K. foliatum* (Pall.) at Wujiaqu, Xinjiang, China (44° 13′ N 87° 40′ E). The GenBank accession numbers for the 16S rRNA gene and genome sequences of strain EGI 63066^T^ are OM691507 and GCA_026241135, respectively.

## Supplementary material

10.1099/ijsem.0.007132 Uncited Supplementary Material 1.

## Abbreviations

ANI: average nucleotide identity
dDDH: digital DNA–DNA hybridization
MA: marine agar
MB: marine broth
MK-6: menaquinone-6
ML: maximum-likelihood
MP: maximum-parsimony
NJ: neighbour-joining
POCP: percentages of conserved proteins
