# Supplementary material for: Galbibacter kalidii sp. nov., a flexirubin-type pigment-producing bacterium, isolated from saline soil in Xinjiang
Source: Int J Syst Evol Microbiol. 2026 May 12;76(5):007132. doi: 10.1099/ijsem.0.007132 (PMC13166967; doi:10.1099/ijsem.0.007132)
Supplement: Uncited Supplementary Material 1. [file ijsem-76-07132-s001.pdf]

Galbibacter kalidii sp. nov., a flexirubin-type pigment-producing bacterium, isolated from saline soil in Xinjiang

Yin Huang[1#], Yibo Yuan[1, 2#], Jinbiao Ma[1], Rashidin Abdugheni[6], Man Cai[3], Yongxia Wang[4], Wen-Jun Li[5], Li Li[1*]

[1] State Key Laboratory of Ecological Safety and Sustainable Development in Arid Lands, Xinjiang Institute of Ecology and Geography, Chinese Academy of Sciences, Urumqi 830011, China.

[2] College of Life Science, Northwest A&F University, Yangling, Shaanxi 712100, China.

[3] China General Microbiological Culture Collection Center, Institute of Microbiology, Chinese Academy of Sciences, Beijing 100101, China.

[4] Yunnan Institute of Microbiology, School of Life Sciences, Yunnan University, Kunming 650091, China.

[5] State Key Laboratory of Biocontrol, Guangdong Provincial Key Laboratory of Plant Resources, School of Life Sciences, Sun Yat-Sen University, Guangzhou 510275, China.

[6] Department of Microbiology, School of Basic Medical Sciences, Xinjiang Medical University, Urumqi 830017, China.

*Corresponding author: Li Li (lili.bobo@outlook.com);

Running title: *Galbibacter kalidii* sp. nov.

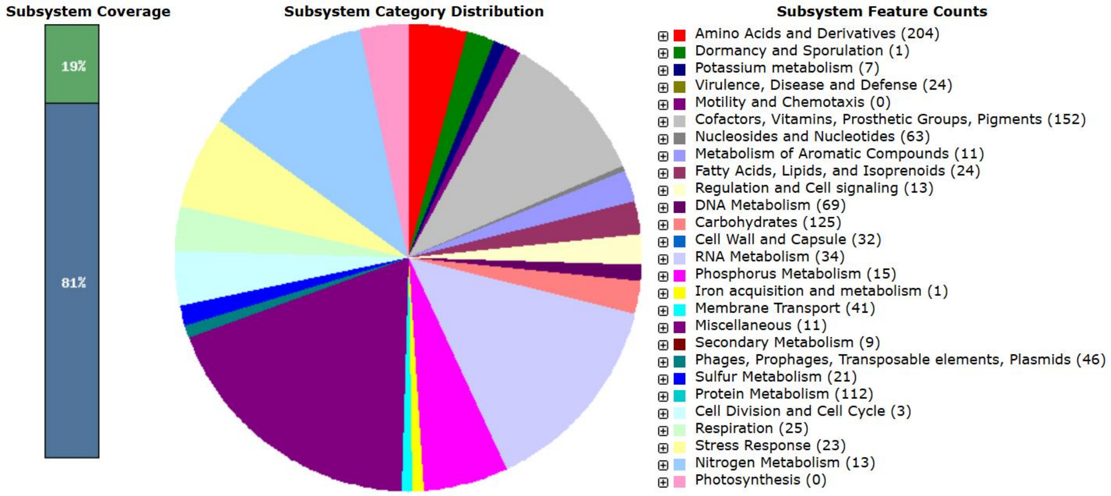

Fig. S1. The genome of strain EGI 63066^{T} was annotated using the RAST server. The green in the left-hand bar chart corresponds to the percentage of proteins included. The pie chart and count of subsystem features in the right panels indicate the percentage distribution and category of subsystems in strain EGI 63066^{T}.

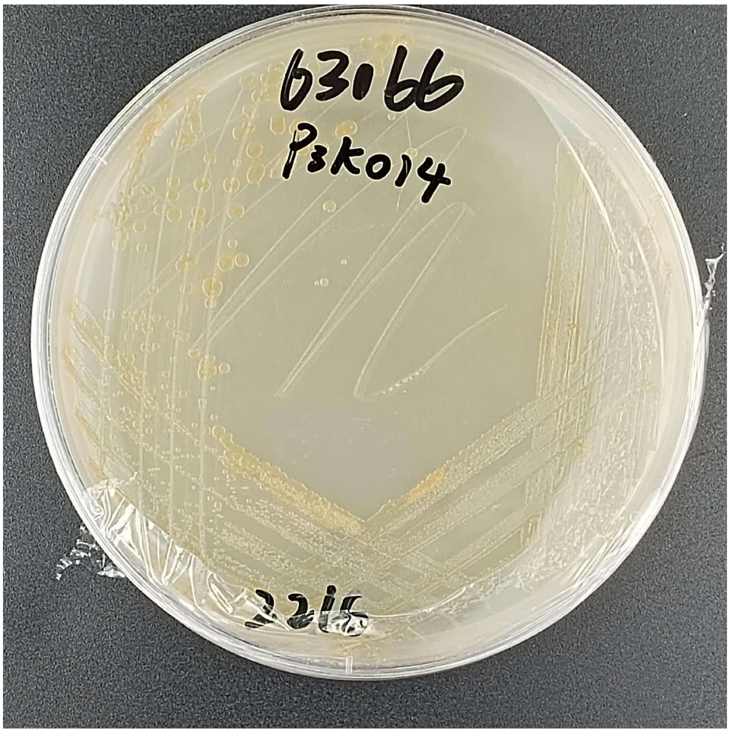

Fig.S2. Colony morphology of the strain EGI 63066^{T}. Colonies were pale yellow, circular, opaque, and smooth after incubation on MA at 30 °C for 48 h.

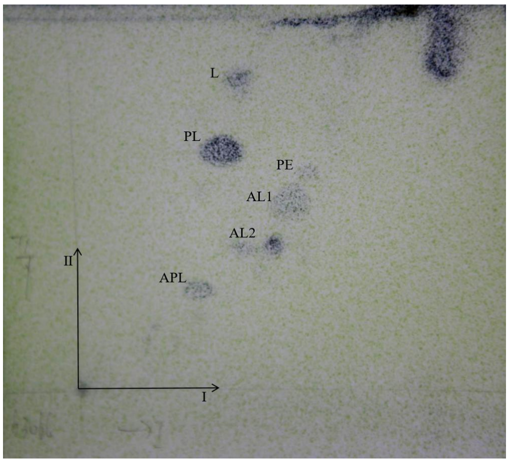

Fig. S3. Polar lipid profiles of strain EGI 63066^T. The TLC plates were stained with molybdophosphoric acid. PE, phosphatidylethanolamine; AL, aminolipid; APL, aminophospholipid; PL, phospholipid; L, unidentified lipids.

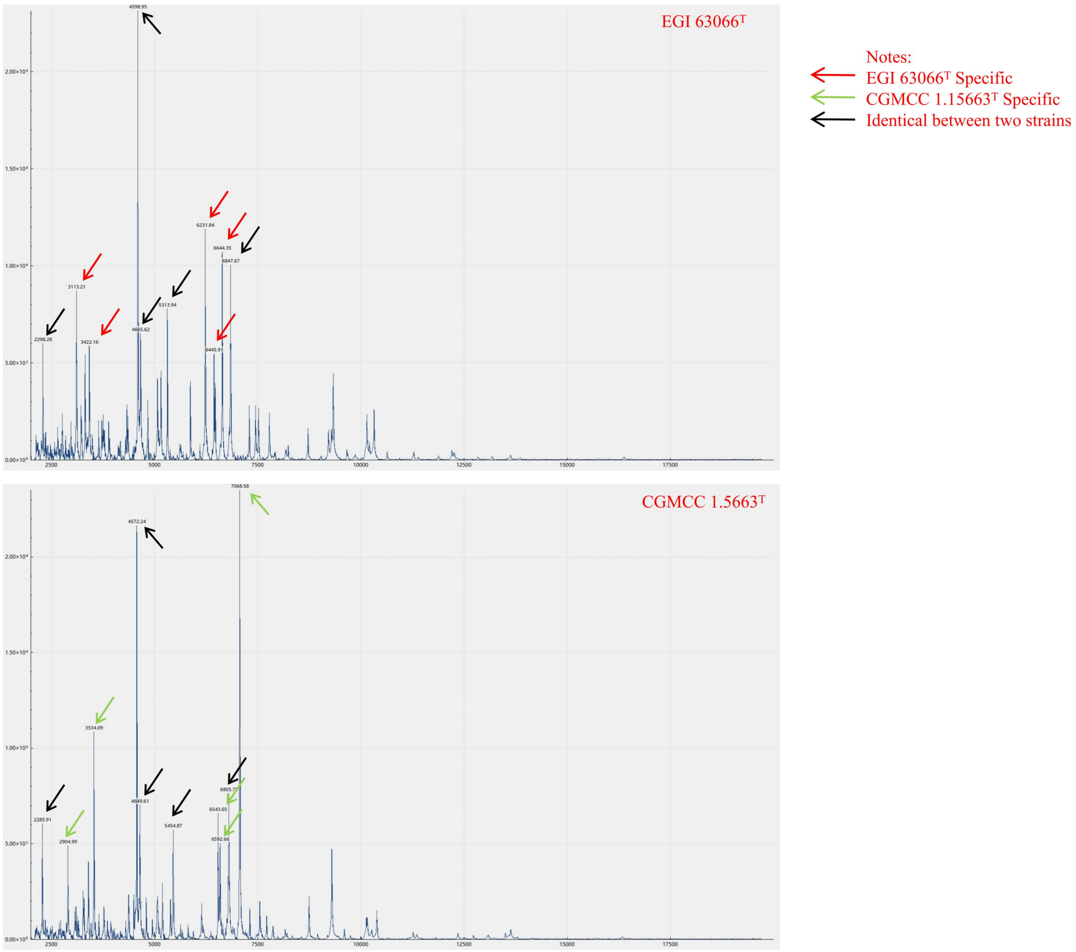

Fig. S4. MALDI-TOF MS spectra analysis revealed the different whole-cell protein profiles of the strain EGI 63066^T compared against *G. mesophilus* CGMCC 1.5663^T.

Table S1. AAI and POCP values between the members of the genera *Galbibacter* and the closest relatives within the family *Flavobacteriaceae*.

AAI (%)	1	2	3	4	5	6	7	8	9	10	11	12	13
1_EGI 63066^T	100	76.7	76.8	72.1	76.9	70.3	69.4	69.8	69.4	68.1	67.5	69.7	69.7
2_*Galbibacter mesophilus* NBRC_101624^T		100	78.5	73.3	80.3	70.5	69.4	69.7	69.5	68.4	68.0	69.3	69.5
3_*Galbibacter pacificus* CMA-7^T			100	74.0	80.2	70.2	69.2	69.6	69.2	67.6	67.9	69.1	69.2
4_*Galbibacter marinus* ck-I2-15^T				100	74.2	68.6	68.0	68.1	67.5	66.6	67.1	67.5	67.5
5_*Galbibacter orientalis* DSM 19592^T					100	69.9	69.0	69.4	69.1	67.7	68.9	69.0	69.2
6_*Robertkochia solimangrovi* CL23^T						100	70.0	70.2	68.0	66.9	66.6	67.7	67.8
7_*Robertkochia sediminum* 1368^T							100	76.9	67.3	67.2	66.9	67.6	67.8
8_*Robertkochia marina* CC-AMO-30D^T								100	67.6	67.2	67.1	67.9	68.1
9_*Abyssalbus ytuae* MT3330^T									100	67.9	67.2	69.2	69.4
10_*Sinomicrobium weinanense* FJxs^T										100	77.5	68.0	68.1
11_*Sinomicrobium oceani* PAP.21^T											100	67.7	67.9
12_*Zhouia amylolytica* HN-171^T												100	81.9
13_*Zhouia spongiae* HN-Y44^T													100
POCP (%)	1	2	3	4	5	6	7	8	9	10	11	12	13
1_EGI 63066^T	100	60.7	64.3	61.4	62.1	56.4	52.6	53.0	54.4	55.3	53.4	56.0	56.7
2_*Galbibacter mesophilus* NBRC_101624^T		100	70.2	64.6	69.4	61.0	61.2	61.6	56.6	51.4	50.4	61.5	60.9
3_*Galbibacter pacificus* CMA-7^T			100	69.4	73.7	63.0	57.3	57.9	57.7	56.2	57.0	60.5	61.8
4_*Galbibacter marinus* ck-I2-15^T				100	68.7	59.3	56.5	56.5	54.8	54.2	55.3	59.5	60.4
5_ *Galbibacter orientalis* DSM 19592^T					100	61.3	57.0	57.5	55.8	54.1	57.4	60.5	61.9
6_*Robertkochia solimangrovi* CL23^T						100	62.1	62.7	56.4	52.6	51.7	59.7	58.2
7_*Robertkochia sediminum* 1368^T							100	80.9	54.4	47.5	45.5	59.5	57.3
8_*Robertkochia marina* CC-AMO-30D^T								100	55.3	47.8	46.0	60.1	57.7
9_*Abyssalbus ytuae* MT3330^T									100	51.4	48.9	55.6	54.9
10_*Sinomicrobium weinanense* FJxs^T										100	67.0	56.2	56.7
11_*Sinomicrobium oceani* PAP.21^T											100	52.2	54.3
12_*Zhouia amylolytica* HN-171^T												100	78.4
13_*Zhouia spongiae* HN-Y44^T													100

Table S2. Isolation sources of strains within the genus *Galbibacter*.

No	Accession number	Organism name	Sequence length (bp)	Isolation source
1	OM691507	*Galbibacter kalidii* EGI 63066	1401	Saline soil
2	AMSG01000057	*Galbibacter marinus* ck-I2-15	1508	Deep sea sediment
3	AB681493	*Galbibacter mesophilus* NBRC 101624	1445	Marine sediment
4	OP862737	*Galbibacter pacificus* CMA-7	1526	Marine surface seawater
5	OM070335	*Galbibacter orientalis* DSM 19592	1419	Marine surface seawater
